# Development of novel composite data quality scores to evaluate facility-level data quality in electronic data in Kenya: a nationwide retrospective cohort study

**DOI:** 10.1186/s12913-023-10133-2

**Published:** 2023-10-23

**Authors:** Beryne M. Odeny, Anne Njoroge, Steve Gloyd, James P. Hughes, Bradley H. Wagenaar, Jacob Odhiambo, Lilly M. Nyagah, Ayub Manya, Ooga Wesley Oghera, Nancy Puttkammer

**Affiliations:** 1 Department of Surgery, Washington University in St. Louis, St. Louis, MO USA; 2 International Training and Education Center for Health (I-TECH), Seattle, WA USA; 3 Department of Global Health, University of Washington, Seattle, WA USA; 4 Department of Biostatistics, University of Washington, Seattle, WA USA; 5 Department of Epidemiology, University of Washington, Seattle, WA USA; 6 The Palladium Group, Nairobi, Kenya; 7 Ministry of Health, Nairobi, Kenya

**Keywords:** EMRs, DHIS2, Data quality assessment, HIV

## Abstract

**Background:**

In this evaluation, we aim to strengthen Routine Health Information Systems (RHIS) through the digitization of data quality assessment (DQA) processes. We leverage electronic data from the Kenya Health Information System (KHIS) which is based on the District Health Information System version 2 (DHIS2) to perform DQAs at scale. We provide a systematic guide to developing composite data quality scores and use these scores to assess data quality in Kenya.

**Methods:**

We evaluated 187 HIV care facilities with electronic medical records across Kenya. Using quarterly, longitudinal KHIS data from January 2011 to June 2018 (total N = 30 quarters), we extracted indicators encompassing general HIV services including services to prevent mother-to-child transmission (PMTCT). We assessed the accuracy (the extent to which data were correct and free of error) of these data using three data-driven composite scores: 1) completeness score; 2) consistency score; and 3) discrepancy score. Completeness refers to the presence of the appropriate amount of data. Consistency refers to uniformity of data across multiple indicators. Discrepancy (measured on a Z-scale) refers to the degree of alignment (or lack thereof) of data with rules that defined the possible valid values for the data.

**Results:**

A total of 5,610 unique facility-quarters were extracted from KHIS. The mean completeness score was 61.1% [standard deviation (SD) = 27%]. The mean consistency score was 80% (SD = 16.4%). The mean discrepancy score was 0.07 (SD = 0.22). A strong and positive correlation was identified between the consistency score and discrepancy score (correlation coefficient = 0.77), whereas the correlation of either score with the completeness score was low with a correlation coefficient of -0.12 (with consistency score) and -0.36 (with discrepancy score). General HIV indicators were more complete, but less consistent, and less plausible than PMTCT indicators.

**Conclusion:**

We observed a lack of correlation between the completeness score and the other two scores. As such, for a holistic DQA, completeness assessment should be paired with the measurement of either consistency or discrepancy to reflect distinct dimensions of data quality. Given the complexity of the discrepancy score, we recommend the simpler consistency score, since they were highly correlated. Routine use of composite scores on KHIS data could enhance efficiencies in DQA at scale as digitization of health information expands and could be applied to other health sectors beyondHIV clinics.

**Supplementary Information:**

The online version contains supplementary material available at 10.1186/s12913-023-10133-2.

## Background

High-quality data from health information systems are imperative to tracking progress toward achieving Joint United Nations Programme on HIV/AIDS (UNAIDS) 95–95-95 targets by 2030 [1]. The UNAIDS 95-95-95 targets are to ensure that 95% of people living with HIV are diagnosed, 95% of those diagnosed are on antiretroviral therapy (ART) and 95% of those on ART are virally suppressed. In Kenya, the expansion of HIV services over the past two decades has been attended by a complementary surge in paper-based registries for documentation of care processes along the HIV care cascade. These processes include HIV testing and diagnosis, linkage to care and anti-retroviral treatment (ART), clinical and virological monitoring, Prevention of mother-to-child Transmission (PMTCT), and infant prophylaxis, among others. Inadvertently, numerous treatment indicators and registries, and their storage locations, have increased the documentation workload shouldered by a severely understaffed healthcare workforce [2–4]. These conditions, alongside other factors like lack of consistent, real-time data entry at the point of service delivery, poor data transmission, duplicate registry of information and infrequent use of data for decision-making, increase the likelihood of error-prone data entry and poor data quality overall [3–6].

In response to the need to bolster data management efforts in HIV treatment facilities, the Kenyan Ministry of Health (MOH), through support from the President's Emergency Plan For AIDS Relief (PEPFAR) began scaling up Electronic Medical Record (EMR) systems from 2012 [7, 8]. By 2019, at least 700 facilities were using an EMR system. Despite the introduction of EMRs, poor data quality poses formidable obstacles to effective data utilization – a challenge that many low- and middle-income countries (LMICs) grapple with [4, 9–13]. Systems for routine data quality assessments (DQAs) were successfully implemented by the Kenyan MOH and partners to monitor and strengthen data quality as EMRs expanded throughout the country [8].

In HIV programs in Kenya, routine DQAs have been instrumental in identifying and resolving data quality concerns while enhancing the data’s usefulness for national health programming [14]. They are vital for accurate evaluation of public health programs and interventions. DQAs assess various elements of data quality including completeness, consistency, and discrepancy (which has to do with plausibility) which map onto data accuracy (see Table 1 for definitions) [14–16]. DQAs can be conducted routinely or periodically as in-person audits and/or remotely using electronic databases at the facility, regional, or national levels [5, 8, 14, 17–27]. In-person audits primarily require physical visits to health facilities to review and cross-check documents [5, 8, 9, 22, 26, 28–31].

**Table 1:** Data quality elements of interest and definitions [14–16]

Data quality element	Definition/Description
Completeness	Refers to “having the appropriate amount of data present.”
Consistency	Consistency refers to uniformity of data across multiple related indicators. For example, number of males and females in care should be equal to number of adults and children in care
Discrepancy	Refers to validation rules defining the possible valid values for the data element, e.g., 1) maximum and minimum acceptable value, and 2) degree of deviation from expected values

Electronic health records and databases, such as the District Health Information System version 2 (DHIS2) have also been used to assess data quality across LMICs [27]. The Kenya Health Information System (KHIS) – which is based on the DHIS2 – is the official MOH data repository in Kenya, and it contains aggregate data on HIV- and non-HIV-related health indicators, with the health facility as the reporting unit. In Kenya, HIV clinics with EMRs can use aggregate EMR data to populate the KHIS. DQAs of the web-based KHIS can be conducted remotely without the need for in-person facility visits or retrieval of paper records. As such, remote DQAs of KHIS can be used to determine the quality of facility-level data used to populate them. While in-person facility-level DQAs are highly detailed and effective, they can be inefficient, costly (e.g., large surveys), cumbersome (involving retrieval of paper records or patient charts), and time intensive for healthcare workers. In-person DQAs typically involve comparing different data records, such as registries, EMRs and/or patient charts, for elements of data quality like consistency, completeness, and reliability among others [14]. These exercises typically require significant person-time to carry out, for example, one DQA in Kenya required teams of 3–4 people, approximately, one day per health facility [8]. These inefficiencies are compounded by contextual factors in LMICs like frequent provider turnover, understaffing, competing workplace demands, the multiplicity of data collection tools and indicators, and lack of provider training in DQA, limited funds, rapidly changing donor priorities, and verticalized programs without centralized data systems [3, 4, 32, 33]. Considering these barriers, in-person assessments may not be feasibly conducted at the scale and frequency needed for growing HIV programs.

For this study, we proposed a retrospective analysis of KHIS data in Kenya to perform DQAs at scale via automated queries as a complement or alternative to time-intensive in-person DQAs. The aims of this research were three-fold: (1) to generate and compare the correlation of three, data-driven composite data quality scores which include a completeness score, a consistency score, and a discrepancy score. These dimensions of data quality were determined a priori and have been shown to be of high importance to health workers [22, 34]; (2) to use these composite scores to identify individual problematic HIV indicators by clinical service department; (3) the 3^rd^ objective of this study was to use composite scores to categorize health facilities into high, fair, or low performing and determine whether the three scores categorized facilities similarly, i.e., facilities would fall into the same rank of high-, fair-, or low-performing regardless of the composite score used to categorize them. We hypothesised that these composite scores would help identify and prioritize areas (indicators and health facilities) for data quality improvement. We hypothesized that the facilities would be consistently ranked into the same categories by all three scores.

## Methods

### Study design and setting

**Fig. 1:**
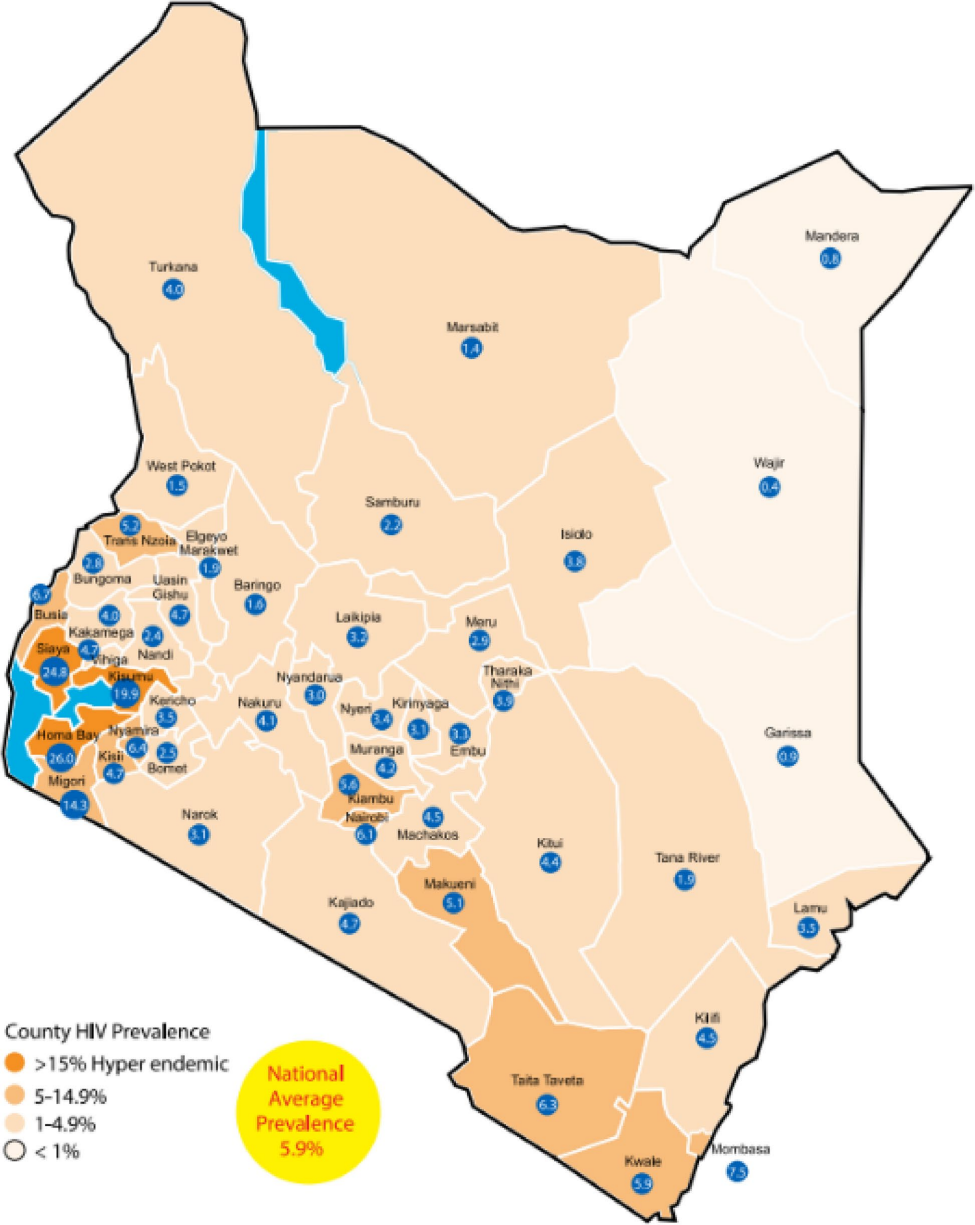
Map of county HIV burden in Kenya—National AIDS Control Council estimates 2018 [35]

This was a retrospective cohort study of KHIS data from 187 facilities implementing EMRs (specifically, KenyaEMR and IQCare EMR platforms) across HIV programs throughout Kenya (Fig. 1). There are three main EMRs used in Kenya supporting HIV programs and we focused on KHIS data from facilities operating two of the most widely used: KenyaEMR and IQCare.

### Sampling

From a pool of approximately 700 facilities with EMRs, we randomly selected 129 health facilities. Additionally, we purposively selected an additional 58 facilities from five high HIV burden counties (Kisumu, Homabay, Siaya, Nairobi, and Migori), for a total of 187 facilities across Kenya. The 58 facilities with EMRs in high burden counties were included a priori, with the knowledge that the heavy investments in HIV programming in these regions would potentially undergird data management processes and lead to better data quality. Investments would potentially consist of staffing, computer hardware, software support, technical, and supervisory support.

### Data source

DHIS2 is an internationally recognized, web-based, open-source platform used by national governments to aggregate, track, and report health facility data [36, 37]. Ideally, health facility personnel use aggregated data from their EMRs to report on health service delivery indicators in the KHIS system, thus KHIS data quality would reflect facility-level data quality. For efficiency, KHIS data can be remotely assessed and evaluated for quality, as a proxy for in-person DQAs.

The KHIS data collected from health facilities is used for monitoring and planning the national HIV program. The data is also used for forecasting and budgeting resources needed by the HIV program. Lack of completeness or inaccuracies in data would mean segments of the population would not be appropriately accounted for in national planning and budgeting.

### Data collection

**Fig. 2:**
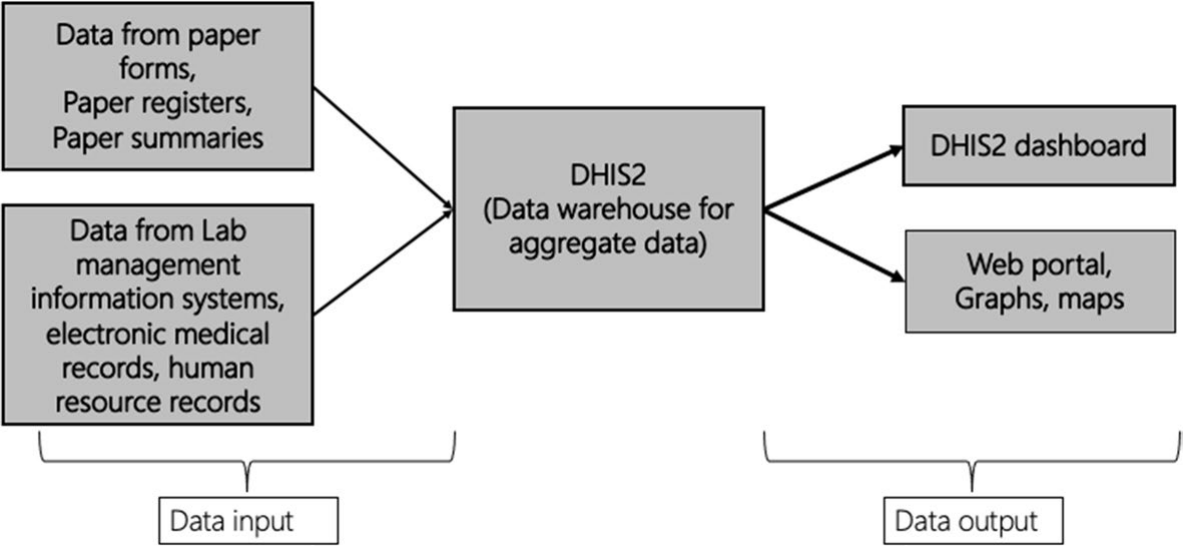
District Health Information System version 2 (DHIS2) flow of information. Created by author, Beryne Odeny

Routinely, each facility generates summary forms, which include aggregated data for various HIV indicators (e.g., number tested, number in HIV care, number on ART etc.), and these are uploaded to the KHIS database. Please see Fig. 2 for a graphic depiction of data flow from point of collection and aggregation to the KHIS.

Data from 187 facilities were extracted from the KHIS database. These data were based on 18 HIV-related indicators. A list of HIV-program indicators available in KHIS is shown in Additional file 1: Appendix 1 (in supplementary material). The data were pulled quarterly from January 2011 to June 2018 and the indicators were used to create the formulas that were termed “data checks” (i.e., logic checks that were used to verify how the variables relate to each other) as listed in Table 2 below. These data checks are what were used in the analysis and generation of composite scores. In total, there were 30 quarters (i.e., from January 2011 to June 2018) and 187 facilities resulting in a total of 5,610 facility quarters (observations).

**Table 2:**
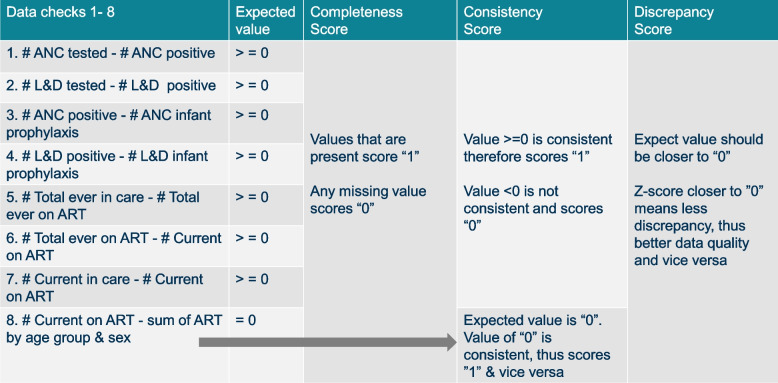
Data checks and composite scores for completeness, consistency, and discrepancy (plausibility)

# Number of people/ individuals

“-” minus (subtraction) sign

*ANC* Antenatal, *L&D* Labor & Delivery, *ART* Antiretroviral therapy

#### Data checks and HIV-related indicators

We used HIV-related indicators – encompassing general adult and pediatric HIV care, antenatal care (ANC), Labor & Delivery care (L&D), and Prevention of mother-to-child Transmission of HIV (PMTCT) – that were uploaded monthly to KHIS. The data consisted of aggregate health service utilization statistics by department. Appropriate data checks were determined a priori, and these checks primarily summarized relationships between indicators to ensure the data were complete, consistent, and plausible. For example, one data check compared the total number of patients in HIV care in a specific quarter versus the total number on ART in that quarter. The difference between the two indicator values was expected to be zero or greater (the logic being that those enrolled would always be more or equal to those receiving ART). A series of data checks were used to construct composite scores for each unique facility. Table 2 below summarizes the data checks and indicators explored in this analysis.

As outlined in Table 2, four ANC and PMTCT data checks (#1 – #4) were computed as differences between related indicator values. Similarly, four general HIV care data checks (#5 – #8) were computed as differences between general HIV care indicator values. The difference for all data checks were expected to be greater than or equal to zero (except data check # 8 which was expected to only be 0, see Table 2).

## Description of composite data quality score generation

Three data-driven composite scores were created as follows:

- 1. Completeness score

The completeness score aims to determine whether aggregate data on the two HIV indicators that constitute a particular data check are present or missing (Table 2). If data on the indicators are missing, it means that individual patient data was not aggregated into the summaries that are uploaded to the KHIS. For example, data check 1 compares two indicators: 1) the number of women tested for HIV in antenatal care (ANC) and 2) the number of women testing HIV positive in ANC. When both values are missing, the data check will miss a value for that specific facility. This is a measure of completeness that looks at two related variables that should both be present.

The completeness score was based on the proportion of data checks which were complete for each observation i.e., each unique facility quarter. A binary score of 1 or 0 was assigned to each data check based on the presence or missingness of the data (1 = present, 0 = missing). The completeness score was a continuous score computed as the proportion (percent) of data checks with complete data for each observation (i.e., unique facility-quarters). The minimum possible score was 0% (0 complete out of 8 checks) and the maximum possible was 100% (8 complete out of 8 checks).

- 2. Consistency score

The consistency score is meant to assess internal consistency of the data by comparing the related indicators of interest against each other. Values of related indicators should track in a similar direction. We use the data checks 1–8, to determine whether the paired HIV indicators are consistent in producing the expected results based on a simple subtraction formula of the count numbers. For example, data check 1 is the “number of women tested for HIV in ANC minus number of women testing positive in ANC.” This number of women tested for HIV in ANC should be greater than or equal to the women who newly test positive for HIV in ANC. The expected value should be greater than or equal to 0. If the observed value is as expected then the data check is consistent and that will be a score of “1” point, but if the observed value is < 0, then the data check is inconsistent, thus the score is “0.”

The consistency score was a continuous score based on the proportion of data checks that were consistent (i.e., scored “1”, among data checks that were not missing). The values of data checks #1–7 were expected to be equal or greater than zero, and that of data check #8 (Total number of patients currently on ART minus the sum of patients on ART across all age groups) was expected to be equal to zero only. On this basis, values for data checks #1–7 which were less than zero (< 0), and data check #8 values which were not zero, were scored “0” for being inconsistent. Conversely, values for data checks #1–7 which were greater than or equal to zero, and data check # 8 values which were equal to zero were designated a score of “1” for being consistent.

- 3. Discrepancy (Plausibility) score

This approach was based on the magnitude of the deviation or discrepancy (as a continuous measure) of observed data check values (Table 2) from the expected value i.e., how far observed values were from zero. Due to differences in facility sizes and patient numbers, deviations from the expected values were standardized using *Z-*score transformations in order to objectively compare the degree of discrepancy [38]. As demonstrated in the explanation of the consistency scores above, data checks #1 -7 have expected values that should be greater than or equal to 0. Data check 8 has an expected value that should be equal to 0, only. For example, for data check 7, “the current number of patients enrolled in HIV care should be greater than or equal the current number of patients receiving ART.” Therefore, the current number in care minus the current number on ART should return a value greater than or equal to 0. All values greater than or equal to 0 were considered plausible. For any values less than 0, we wanted to measure their degree or extent of deviation in the negative direction when the values were clearly implausible, i.e. below 0. We used *Z*-scores to determine the extent of variation of the discrepant observations from the expected “0” so that we could have a gradation to the deviation/ discrepancy. Higher *Z*-scores meant greater discrepancy or deviation in negative (implausible) values, thus poorer data quality. Lower *Z-*scores meant less discrepancy, thus better data quality. The composite discrepancy score was computed as an average of all the individual *Z-*scores for each unique facility quarter. Further description of the methodology is provided in Additional file 1: Appendix 2.

## Statistical analysis

Descriptive statistics were used to summarize baseline characteristics. Categorical variables were summarized as counts and percentages. Continuous variables such as the composite scores were summarized with means (standard deviation) and medians (interquartile range). A descriptive bar graph and map were used to illustrate the extent of missing data across the data checks and highlight the most incomplete data. To determine how similar or correlated the three score profiles were, the Spearman rho correlation test was used. Panel plots of individual Z-scores for discrepancy were used to identify the high and low performing HIV indicators by service department.

For each of the three scoring profiles, facilities were ranked as high-, fair-, or low-performing. These categories were defined using percentile cut-offs; those below the 30^th^ percentile were considered low performing, those between the 30^th^ and 60^th^ percentile were considered fair performing, and those above the 60^th^ percentile were considered high performing. All statistical analyses for this evaluation were done using R studio version 3.6.2 (2019–12-12).

## Ethics approval and consent to participate

The protocol was reviewed by the University of Washington’s Human Subjects Division which determined that the evaluation did not involve human subjects, as defined by federal and state regulations. Therefore, ethical approval by the University of Washington Institutional Review Board was not required. This is not a study reporting experiments on humans and/or the use of human tissue samples. We used national, aggregated, routine health data (non-research data) that was de-identified and fully anonymized before we accessed them, thus the ethics committee waived the requirement for informed consent. The need for informed consent was waived by the University of Washington Institutional Review Board and the African Medical and Research Foundation (AMREF) Ethical Scientific Review Committee. The United States (US) CDC’s Center for Global Health Office of the Associate Director for Science (ADS) approved the protocol (#2018–528) and local IRB clearance was granted by the AMREF Ethical Scientific Review Committee.

All methods were performed and reported in accordance with the “Strengthening the Reporting of Observational Studies in Epidemiology (STROBE)” guidelines.

All methods were performed in accordance with the relevant guidelines and regulations for the publication of non-research, observational routine health data.

## Results

### Baseline characteristics

Characteristics of facilities are summarized in Table 3.

**Table 3:** Baseline characteristics of health facilities based on 2017 facility survey

*N* = 187 facilities	
**Facility characteristics**	**N (%)**
Facility type	
County hospital	4 (2)
Sub-county hospital	69 (37)
Health center	77 (41)
Dispensary	37 (20)
High-volume facility (> 500 patients)	
Yes	118 (63)
No	69 (37)
EMR type	
KenyaEMR	112 (60)
IQCare	75 (40)
High HIV-burden county	
Yes	58 (31)
No	129 (69)

County hospital: These are county referral hospitals which offer more specialized services and have a higher bed capacity compared to sub-county hospitals

Sub-county hospital: Typically run by medical doctors and additionally, have a surgery unit

Health center: Typically run by clinical officers and offer in-patient services serving a larger catchment population compared to dispensaries

Dispensary: Run and managed by registered nurses and only provides outpatient services for minor ailments

### Summary of composite scores

The mean completeness score was 61.1% [standard deviation (SD) = 27.0%], illustrating that on average, approximately sixty percent of individual data checks were complete. Of 5,610 observations, 953 were missing consistency scores and discrepancy scores because they were missing all data check values for that facility-quarter (Table 4). The mean consistency score was 80% (SD = 16.4%), illustrating that an average eighty percent of complete data checks were consistent. The mean discrepancy (plausibility) score was 0.07 (SD = 0.22). Please see Table 4.

**Table 4:** Descriptive statistics of composite scores by approach

		Total *N* = 5,610			
Score approach	Mean (SD)	Median (IQR)	Minimum	Maximum	Missing N (%)^d^
Completeness score^a^	61.1 (27.0)	75.0 (50.0 -75.0)	12.5	100	N/A
Consistency score^b^	80.0 (16.4)	83.3 (66.7–100)	0	100	953 (17)
Discrepancy score^c^	0.07 (0.22)	0.02 (0—0.06)	0	6.16	953 (17)

*SD* Standard Deviation, *IQR* interquartile Range

^a^Completeness Score (%) is based on the proportion of data checks which are complete for each facility by quarter

^b^Consistency score (%) was derived by calculating the proportion of complete data checks that had consistent data

^c^Discrepancy score has a lower bound of 0 and is based on *Z-*scores which depict degree of discrepancy with expected values

^d^Refers to the number of unique facility quarters that had missing values for all 8 data checks

### Missing data

**Fig. 3:**
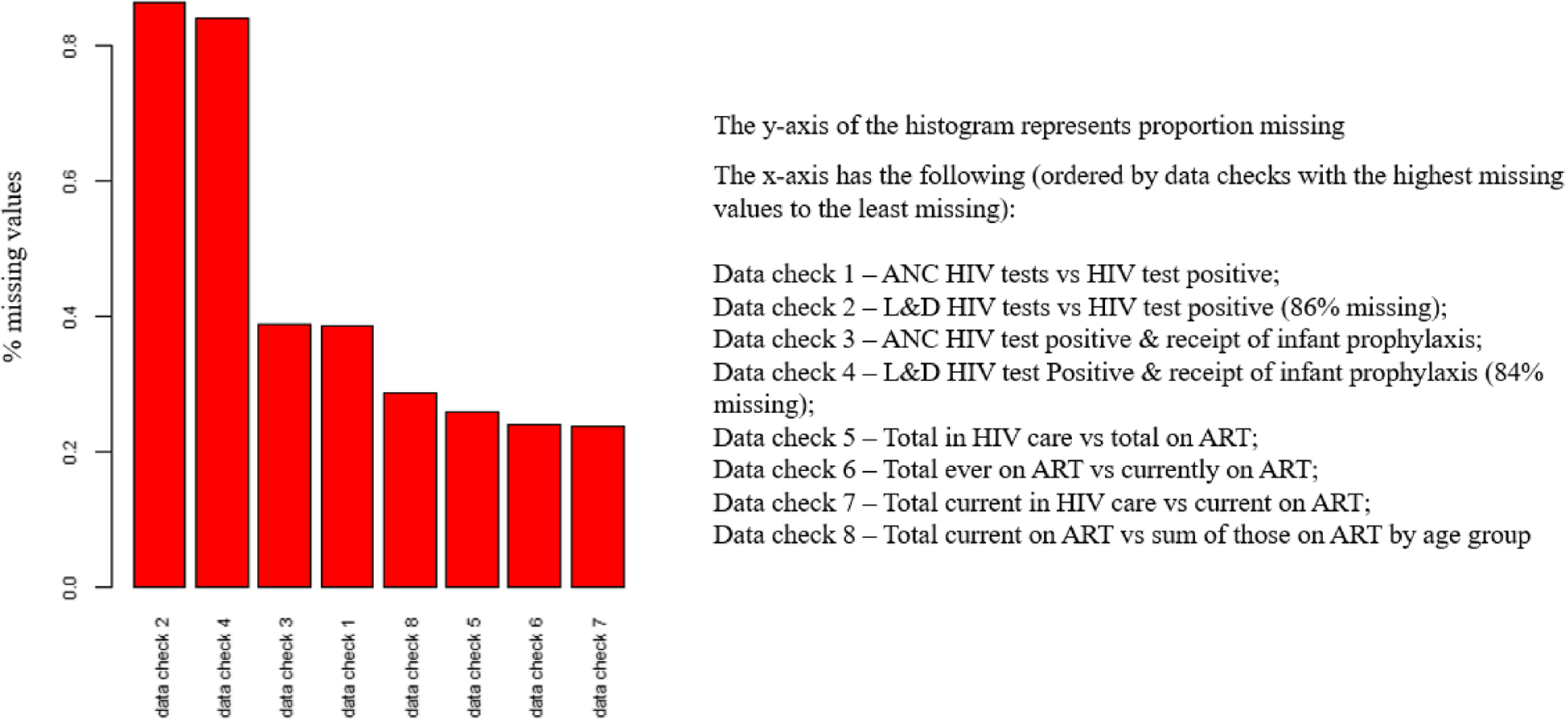
Percent of missing data for each data check. ANC – Antenatal care; L&D – Labor and delivery department; ART – Antiretroviral Therapy; HIV – Human Immunodeficiency Virus

Figure 3 is a bar graph and map of the missing data checks. The bar graph is ordered by the magnitude of missing data across data checks. Labor and delivery HIV testing, positivity, and infant prophylaxis data checks (#2 and #4) were the most incomplete, whereas general HIV care data checks had the lowest proportions of missing data (#5, #6, #7, #8).

### Correlation results

**Fig. 4:**
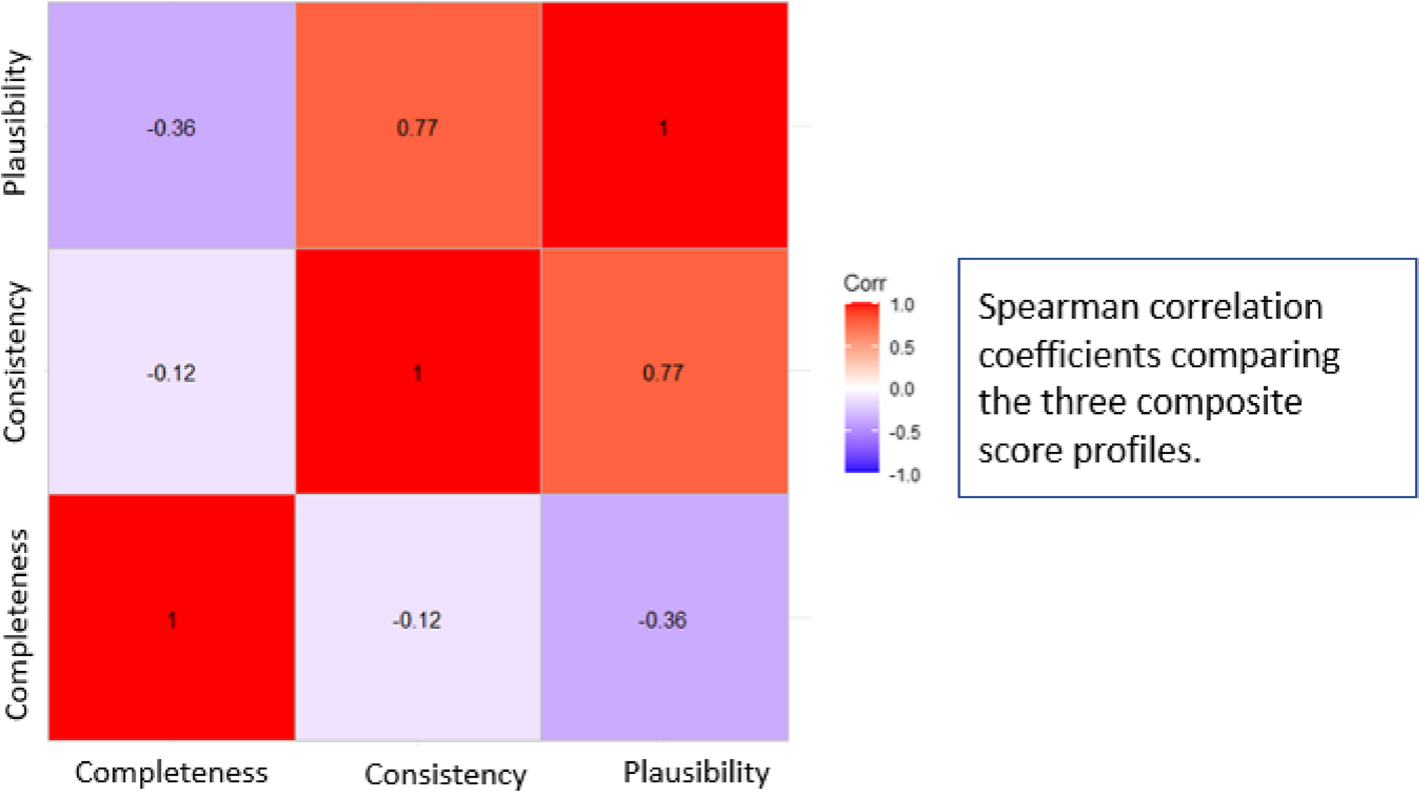
Correlation of the three composite score approaches

The correlation plot below (Fig. 4) illustrates the strength of the correlation coefficients between the different composite scores. The completeness score was weakly correlated with the consistency score and the discrepancy (plausibility) score in the negative direction with correlation coefficients of -0.12 and -0.36, respectively (Fig. 4). On the other hand, the consistency score and the discrepancy (plausibility) score were strongly and positively correlated (correlation coefficient = 0.77).

### High and low performing data checks

**Fig. 5:**
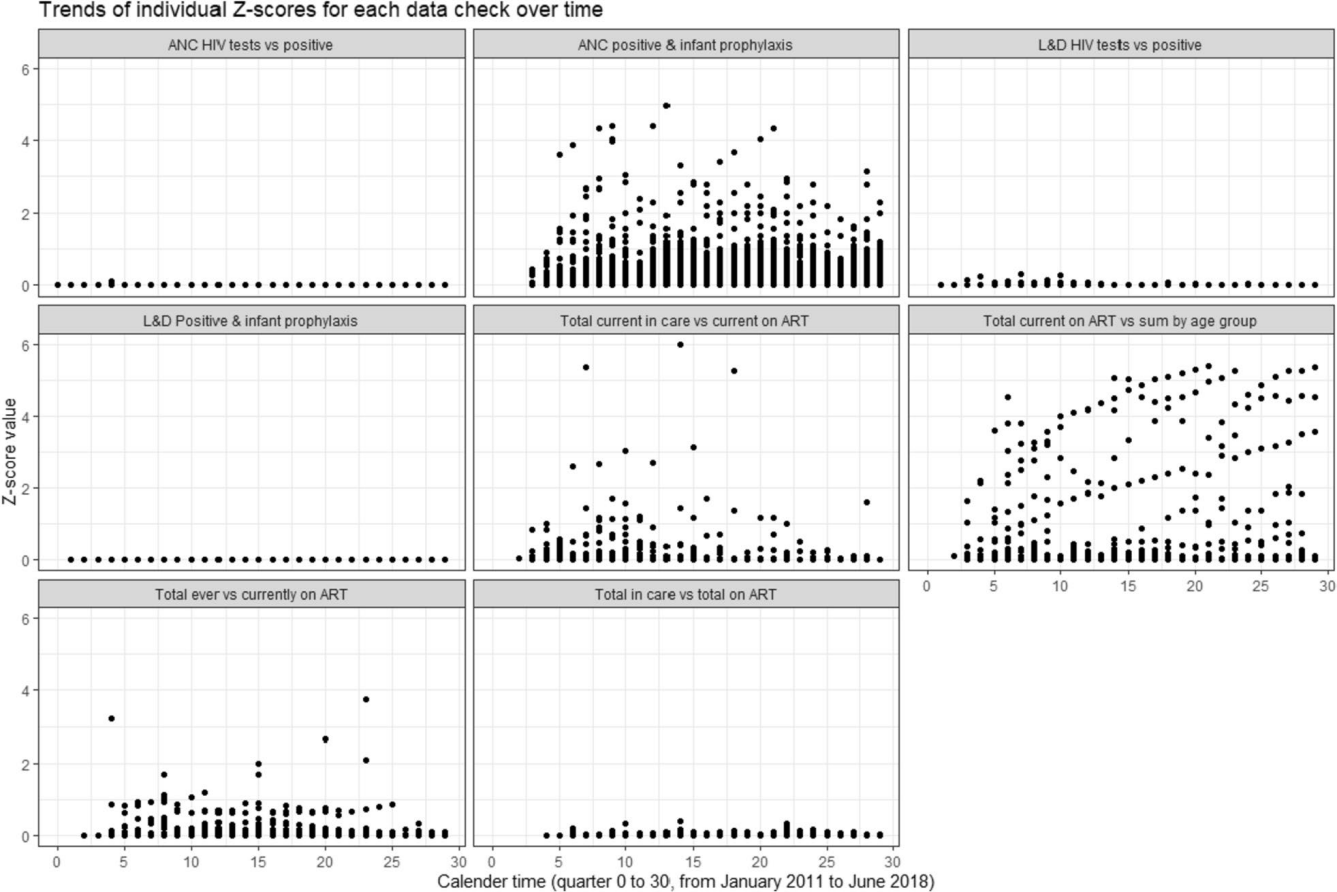
Trends of individual *Z*-scores for discrepancy

Figure 5 provides an overview of the discrepancy (plausibility) assessment using individual *Z*-scores and illustrates the distribution of individual scores over time. Z-scores were capped at a minimum of zero and larger/positive values were associated with greater discrepancy and deviation from expected values, thus poorer data quality and, potentially, clinical care. Most ANC- and L&D-related data checks (# 1, # 3, # 4) and one general HIV care data check # 8 (see Table 3 above) had the most favorable discrepancy (plausibility) scores, i.e., lower values closer to zero. On the other hand, most general HIV care data checks (# 5, # 6, and # 7) and one ANC data check #2 had unfavorable discrepancy (plausibility) scores with considerably larger discrepant values.

The scatter plots in Fig. 5 illustrate the varying degrees of discrepancy for individual data check values over time.

Table 5 provides a summary of facility ranking across all quarters by each composite score profile. While ranking was based on percentiles cut offs, the actual proportion of unique facility quarters in each of the three ranks was not commensurate to the expected proportion (i.e., ~ 30%) in each percentile rank because some scores clustered at the cut off points. For example, if some scores clustered at the 30^th^ or 60^th^ percentile, this translated to fewer scores being in the < 30^th^ or > 60^th^ percentile ranks, and so forth. We also noted considerable uniformity, across the three composite scores, in ranking/ categorization of facilities into high-, fair-, or low- performing. More than 90% of facility observations ranked in the same category across at least two composite scores and > 50% ranked in the same category across all three composite scores (view Table 6 for details). Only four percent (4%) ranked differently across all the scores.

**Table 5:** Summary of facility ranking (across all quarters) by composite score

		Ranking by composite score (N/%)		
		**Completeness** (*N* = 5610)	**Consistency** (*N* = 4,657)	**Discrepancy** (*N* = 4,657)
**Performance** **N (%)**	**High**	736 (13)	1221 (26)	1309 (28)
	**Fair**	3268 (58)	2861 (61)	2904 (62)
	**Low**	1606 (29)	575 (13)	444 (10)

N = unique facility quarters

Ranking is based on cut offs at the 30^th^ and 60^th^ percentile for each composite score. Values below the 30^th^ percentile score are considered low performing. Values between the 30^th^ & 60^th^ percentile are considered fair performing, while those above the 60^th^ percentile are high performing

**Table 6:** Uniformity of ranking (high, fair, or low) across various score profiles (*N* = 5,610)

	All facility quarters N (%)
Uniform ranking across three composite scores	1,805 (32)
Uniform ranking across two composite scores	3602 (64)
Does not rank consistently across any of the scores	203 (4)

## Discussion

Kenya has a rapidly expanding national HIV program that relies on the KHIS system to track, monitor, and evaluate programmatic progress. In this study, KHIS proved to be a convenient and readily available source of nationwide data for DQA. We successfully developed a systematic approach for examining large volumes of data by computing three data-driven composite scores reflecting completeness, consistency, and discrepancy (plausibility) which were instrumental in determining overall data quality, and identification of high-, fair-, or low-performing facilities. Individual completeness, consistency, and discrepancy (plausibility) scores – focusing on individual data checks – were used to detect high versus low performing indicators.

We discovered a low and slight negative correlation between the completeness score and the other two consistency and discrepancy (plausibility) scores. This lack of correlation illustrates that completeness assessment should be paired with measurement of either the consistency or discrepancy (plausibility) to reflect distinct dimensions of data quality. Contrary to our pre-specified analysis plan, we were unable to combine the completeness dimension and the consistency or discrepancy (plausibility) dimensions into one composite metric, for two reasons. First, the high degree of incomplete data would inadvertently be the primary driver of the overall score and give little weight to the other two dimensions. Second, the option of considering a weighted composite score, which combined the three dimensions, was not appropriate given the negative correlation between the completeness score and both consistency and discrepancy (plausibility) scores. We opted to explore these dimensions separately.

The completeness score was strikingly low with 40% missing data on average. The missing data graph revealed missingness as primarily driven by incomplete ANC- and L&D-related data check values. This graph could be used to chart low performing indicators to bolster precise targeting of service departments that need strategies to improve completeness. In contrast with current literature, smaller scale studies have reported higher levels of completeness of data in maternal child health indicators including PMTCT, and general HIV clinics in sub-Saharan African (SSA) [21, 39]. There are several studies from similar contexts which have findings on completeness of maternal child health data. For example, studies in Malawi and South Africa have demonstrated relatively high completeness of HIV data. In Malawi, a study by O’Hagan et al. demonstrated completeness was high across service departments including HIV testing and counselling, though data accuracy varied across service areas [33]. A study of 57 South African facilities by Nicol et al. found completeness levels as high as 96% in PMTCT indicators like infant and maternal HIV testing, and administration of prophylaxis and ART [39]. In a small study of DHIS data by Garrib et al., from 10 clinics in South Africa, clinic data were 97% complete [2]; however, another study in South Africa by Jamieson et al., demonstrated significant variability across facilities – ranging from 22 to 89%) for various HIV and TB treatment indicators [40]. In contrast, a study in Ethiopia by Abiy et al., found intermediate completeness of 76% for HIV EMR data [9], and other settings found low completeness level, such as a study of PMTCT performance in Cote d'Ivoire found that high degrees of missing data [41]. Another study in Uganda found 30% missing data on ART among other indicators [42].

Our nationwide evaluation consists of a larger, longitudinal sample and thus offers a broader snapshot of the level of data completeness in Kenya – capturing both high and low performing areas. Of note, this may be an underestimate of the level of completeness of facility-based paper registers as paper registers are more likely to be complete compared to EMR or KHIS summaries. Paper-based registries are potentially more likely to have a higher completion rate than EMR data. This is because health providers typically use paper records as their primary method of documentation. Few health facilities have point-of-care use of EMRs and most rely on retrospective digital data capture from paper records. Because paper records are the first point of data capture, they are likely to have more information.

The main data collection steps for KHIS involve: 1) data collection from facility charts or registers to generate facility aggregate summary forms; and 2) uploading or data entering data from the aggregate summary forms into KHIS. Lack of complete data in these steps, will manifest, in KHIS as underestimates of count data or missing facility data for specific HIV indicators (such as number cumulatively in HIV care, number currently on antiretroviral therapy (ART), number of males/ females children/ adults receiving ART etc.) Further, this may be associated with lack of facility summary data for a particular quarter, thus lack of facility representation in national reports. This may lead to deficiencies in budget planning and suboptimal forecasting of resource needs for facilities with missing data or underestimated counts for specific indicators. Completeness is equally important at all collection points as any error at one point spills over to the next – from registers to summary forms, and from summary forms to the KHIS.

In comparison to the completeness scores, the consistency and discrepancy scores we observed in this evaluation performed better. We found an average of 80% consistency of complete data checks. Furthermore, the mean discrepancy score suggested that most scores were within 1 SD of the expected value. These high scores of consistency and discrepancy (plausibility) map on to the broader concept of accuracy and may be comparable to high performing reliability and concordance measures examined in other settings [21, 26]. For instance, a study by Endriyas et al. of 163 facilities in Ethiopia found that approximately 85% of facilities reported maternal child health data that was accurate or within the acceptable range [26].

In our evaluation, most individual discrepancy (plausibility) scores for the general HIV care data checks indicated higher levels of discrepancy than ANC- and L&D-related data. This finding, that general HIV indicators were more complete, but less consistent and plausible than PMTCT indicators, underpins the need for in-depth exploration of the reasons behind disparate performance across departments in order to tailor interventions appropriately. A mixed picture of high completeness with low accuracy (depicted by consistency and concordance), and vice versa, was observed when exploring EMR or DHIS2 data in other SSA contexts including Malawi, Ethiopia, and South Africa [2, 9, 13, 26, 33, 39]. In South Africa, the accuracy of PMTCT data, based on concordance across various databases and organizational levels, ranged from 51 to 84% [39]. Contrary to this finding of disparity between completeness score and consistency or discrepancy (plausibility) performance, an assessment of four-year ANC data from 495 facilities in Rwanda, revealed high performance for both completeness and consistency across data sources (absence of extreme outliers) at 98% and 83%, respectively [21]. A study in South Africa revealed relatively high accuracy and concordance of data in HIV/TB clinics ranging from 85–88% accuracy [40]. While the measures used in various studies are not similar or comparable, they provide a foundation for understanding data quality performance across SSA. Our evaluation continues to broaden this understanding as studies from other settings may be disadvantaged by shorter follow-up periods and/or small sample sizes.

With regard to uniformity, the three composite scores were considerably uniform in their ranking of facilities into high, fair, or low performing categories. A third of all facilities maintained their rank – high, fair, or low – across the three scores. Most facilities maintained their rank across at least two of the scores, primarily consistency and discrepancy. This uniformity supports the use of one of the scores to rank facility performance. We recommend the simpler consistency score over the more complex discrepancy score.

## Strengths and limitations

Regarding feasibility, we demonstrate that it is possible to use composite scores to analyze the quality of large volumes of data remotely – this may be more efficient than in-person DQAs. These composite scores are instrumental, not only with timely flagging of low-performing facilities for targeted interventions, but also bring attention to high-performing facilities from which program managers could learn. This study had several strengths beyond efficiency. One of the strengths was the large sample and longitudinal design which allowed repeated measurements of data quality over an eight-year time frame – an added advance over studies that have assessed the data quality cross-sectionally or over shorter timeframes [2, 9, 22, 26, 33]. Further, the use of locally accessible programmatic data adds to the relevance of this study to the Kenyan context, specifically and may thus instigate policy or practice changes that improve routine health information systems in the country. Finally, with regard to external validity, the methodology used in this evaluation can be reused in other countries that use the DHIS2 system [36].

This study had some limitations. We noted higher levels of consistency and discrepancy (plausibility) in data checks that had less complete data. This could have been driven by systematic bias as less complete data would mean less chances to be discrepant. We were unable to compare this KHIS-based DQA with facility-based EMR or paper registry DQAs to give a holistic picture of the data quality landscape. We recommend using KHIS DQA for expeditious identification of facilities that need more intense DQAs which incorporate facility-level DQAs. Another limitation is a multiplicity of EMRs used throughout Kenya and we focused on KHIS data from facilities operating two of the most widely used: KenyaEMR and IQCare. This would limit the generalizability of our findings to reflect facility-level data quality in settings using other less commonly used EMR platforms or paper-based data systems. Due to lack of similarity of metrics across studies, it was not possible to directly compare our findings with those from other settings. We also primarily focused on HIV-related indicators which would reduce the application of our findings to other health service departments. We recommend future expansion of these composite scores to assess data quality in other service departments beyond HIV care.

These composite scores are designed to be simple and efficient at scanning for stark data quality issues that need urgent attention and investigation. Further, high favorable scores may not guarantee accurate data (since data that is “too perfect” could be suspicious), thus outliers with extremely weak and/ or extremely strong scores could be investigated more closely. Given the decline in funding of HIV programs and poor funding available to most non-HIV primary health programs, and the increasing volume of patients and data, the composite scores provide rapid and simple data-driven approaches to assess data quality. We recommend that national policy makers and managers use this approach to screen and rapidly identify problematic sites, for which a deeper granular analysis of data quality can be done. Indeed, assessing individual indicators separately will help distil the exact data points that need remediation.

## Conclusions

Routine DQAs are essential for optimizing RHIS and efficient mechanisms for conducting assessments are urgently needed. In this evaluation, we demonstrate that national-level data can be harnessed to rapidly assess facility-level data quality using a composite scoring system. For a holistic DQA, data completeness score assessment should be paired with measurement of either the consistency or discrepancy (plausibility) score to reflect distinct dimensions of data quality. As health systems – including routine information systems – weather the challenges of ongoing pandemics, routine use of composite scores on DHIS2 data may be a feasible approach to monitoring and upholding data quality in LMICs.

### Supplementary Information

**Additional file 1: Appendix 1.** HIV-indicators captured in DHIS2. **Appendix 2.** Development of composite discrepancy (plausibility) score using z-scores.

## Data Availability

The datasets used and/or analyzed during the current study available from the corresponding author on reasonable request.
