# Supplementary material for: Development of novel composite data quality scores to evaluate facility-level data quality in electronic data in Kenya: a nationwide retrospective cohort study
Source: BMC Health Serv Res. 2023 Oct 23;23:1139. doi: 10.1186/s12913-023-10133-2 (PMC10594801; doi:10.1186/s12913-023-10133-2)
Supplement: Supplementary file 1 — Additional file 1: Appendix 1. HIV-indicators captured in DHIS2. Appendix 2. Development of composite discrepancy (plausibility) score using z-scores. [file 12913_2023_10133_MOESM1_ESM.pdf]

OTHER IMPORTANT SUPPLEMENTARY INFORMATION

APPENDIX 1: HIV-indicators captured in DHIS2

DHIS2 HIV-related indicators

HIV care & treatment

Aggregated numbers:

- Total ever (cumulative) in care
- Total currently in care
- Total currently ART
- Total ever on ART
- Total starting ART
- Currently on ART (female >= 15 years)
- Currently on ART (female <15 years)
- Currently on ART (male >= 15 years)
- Currently on ART (male <15 years)

PMTCT

Aggregated number of patients:

- Antenatal testing for HIV
- Antenatal positive for HIV
- Labor & delivery (L&D) testing for HIV
- L&D positive for HIV
- Postnatal testing for HIV
- Postnatal positive for HIV
- Issued infant prophylaxis (ANC)
- Issued infant prophylaxis (L&D)
- Issued infant prophylaxis (postnatal)

APPENDIX 2: Development of composite discrepancy (plausibility) score using z-scores

This approach utilized *Z*-scores to estimate the extent of deviation or discrepancy of observed values i.e., how far observed values were from expected values. To allow for comparison of values across facilities with varying patient volumes, we standardized the data checks by dividing them by respective facility patient volumes (i.e. general HIV care and treatment data checks were divided by the "total number of patients on ART", and ANC and PMTCT data checks were divided by the "total number of women accessing antenatal HIV testing"). Due to heterogeneity across facilities over time, values were standardized using *Z*-score transformations so that a common scale was used to compare degree of discrepancy [38]. Sites with greater discrepancy of observed values were penalized more than those with less discrepancy. The untransformed unadjusted Z-score was defined as:

$$z = (y - 0)/s_D$$

where y is the observed 'standardized data check value', 0 is the expected value if there is no difference between indicators, and S_D is the standard deviation of y. If the observed data check values are centered around 0, then z has mean 0 and SD 1. We assumed a normal distribution of data check values centered around the difference value, 0, the threshold for discrepancy (plausibility) and good care. An individual discrepancy (plausibility) *Z*-score was computed for each data check and the S_D was based on data from all facilities and quarters.

The composite discrepancy (plausibility) score was computed as an average of all the individual *Z*-scores for each unique facility quarter. Missing data check values were dropped from Z-score computation because the goal was to assess discrepancy (plausibility) of available values regardless of completeness. The discrepancy (plausibility) score was a continuous variable, centered on zero. For data check # 1-7, composite scores >= 0 were considered plausible and accurate regardless of how large or small the positive values were, thus all positive scores (> 0) were assigned a *Z*-score of 0. For data check # 8, values > 0 and values < 0 were both considered implausible. For consistency of handling deviations in the negative and positive direction, all negative individual *Z*-scores were multiplied by a factor of -1 and thus converted to the positive scale. As such, the lowest possible *Z*-score value was "0". Increasing *Z*-scores meant greater discrepancy or deviation of observed values, thus poorer data quality with regard to discrepancy (plausibility). The lower *Z*-scores were associated with less discrepancy, thus better data or greater discrepancy (plausibility). This approach was less stringent than the consistency score as discrepant values which did not considerably deviate away from the expected value were not excessively penalized compared to larger negative values or outliers.
